# Case Report: Transcatheter occlusion of a rare pulmonary artery to left atrium fistula using an atrial septal defect occluder device

**DOI:** 10.3389/fcvm.2025.1698642

**Published:** 2026-03-02

**Authors:** Twalib Olega Aliku, Nestor Mbabazi, Bernard Obongonyinge, Judith Namuyonga, Matthew Jones, Sulaiman Lubega, Shakeel A. Qureshi

**Affiliations:** 1 Department of Paediatric Cardiology, Uganda Heart Institute, Mulago Hospital Complex, Kampala, Uganda; 2 Department of Paediatrics and Child Health, Uganda Christian University School of Medicine, Mukono, Uganda; 3 Department of Paediatrics and Child Health, Mulago National Specialized Hospital, Kampala, Uganda; 4 Department of Paediatrics and Child Health, Makerere University College of Health Sciences, Kampala, Uganda; 5 Department of Paediatric Cardiology, Evelina London Children’s Hospital, Guy’s and St Thomas NHS Foundation Trust, London, United Kingdom

**Keywords:** ASD occluder, cyanosis, device occlusion, fistula, left atrium, pulmonary artery

## Abstract

A right pulmonary artery to left atrium fistula represents a rare cause of cyanosis in childhood. It may be diagnosed late due to the presence of minimal symptoms. We report the case of a 3-year-old child who presented with dyspnea and cyanosis. Transthoracic echocardiography revealed an anomalous fistulous connection between the right pulmonary artery and a large aneurysm communicating with the roof of the left atrium. The patient underwent successful transcatheter occlusion using an atrial septal defect occluder device. The patient had an uneventful recovery on follow-up with resolution of cyanosis.

## Introduction

A pulmonary artery to left atrium (PA–LA) fistula is a rare cyanotic congenital heart defect (CHD). Since the initial description by Friedlich et al. in 1950 (1), fewer than 100 cases have been reported in the literature. Untreated cases may result in hyperviscosity syndrome due to chronic hypoxemia, brain abscess, cerebrovascular accident, systemic thromboembolism, endarteritis, or fatal rupture (2, 3). We describe the case of a 3-year-old boy who presented with dyspnea and cyanosis and subsequently underwent successful transcatheter occlusion of a right pulmonary artery (RPA) to LA fistula using an atrial septal defect (ASD) occluder device.

## Case report

A 3-year-old boy was referred to the Uganda Heart Institute (UHI) with complaints of dyspnea, easy fatigue, and cyanosis. At the time of referral, he was undergoing treatment for severe acute edematous malnutrition. On presentation, he presented with delayed gross motor milestones, as he had not walked until the age of 2 years and 3 months. Clinical examination revealed central cyanosis and grade 3 digital clubbing. Oxygen saturation was 70%. His weight was 14.8 kg and height 118 cm (W/H Z-score ≤ −3SD). Cardiac examination revealed that apex was in the 5th intercostal space, mid-clavicular line, with normal first and second heart sounds and a soft systolic murmur audible over the right posterior chest wall. His hemoglobin was 14 g/dL with a mean cell volume (MCV) of 63 fL, suggesting iron deficiency anemia. Renal and liver function tests were normal. Viral serologic tests for HIV, hepatitis B, and hepatitis C were negative.

**Figure 1:**
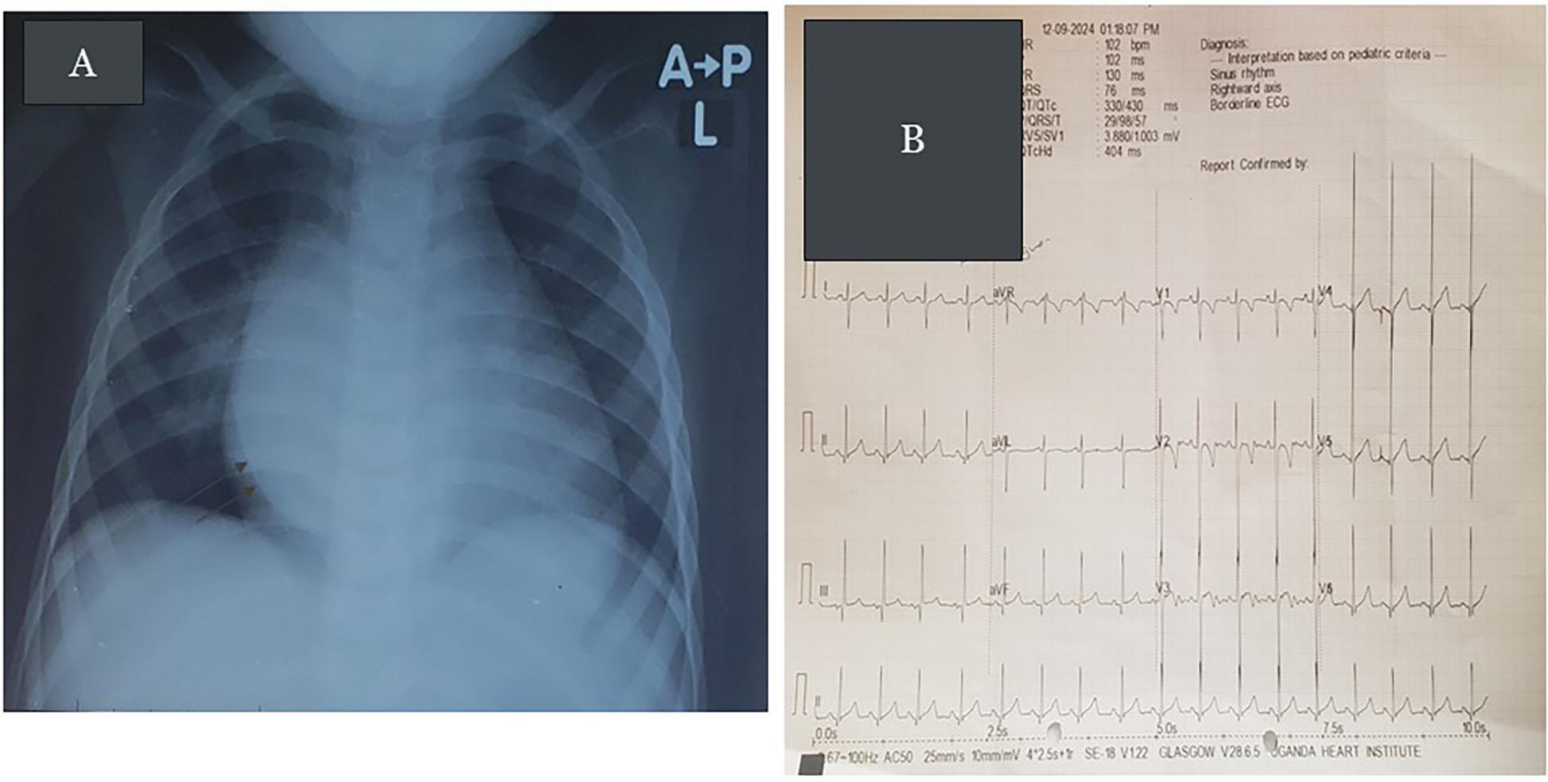
**(A)** Chest radiograph showing cardiomegaly with a double density within the cardiac silhouette on the lower right half, merging superiorly with the right pulmonary artery shadow (eparterial bronchus). There is no splaying of trachea. The features are consistent with the aneurysmally dilated entry zone of the RPA to LA fistula. **(B)** The ECG of the patient showing sinus rhythm with right axis deviation and large equiphasic complexes in v3 (Katz–Watchel phenomenon), suggestive of combined ventricular hypertrophy.

**Figure 2:**
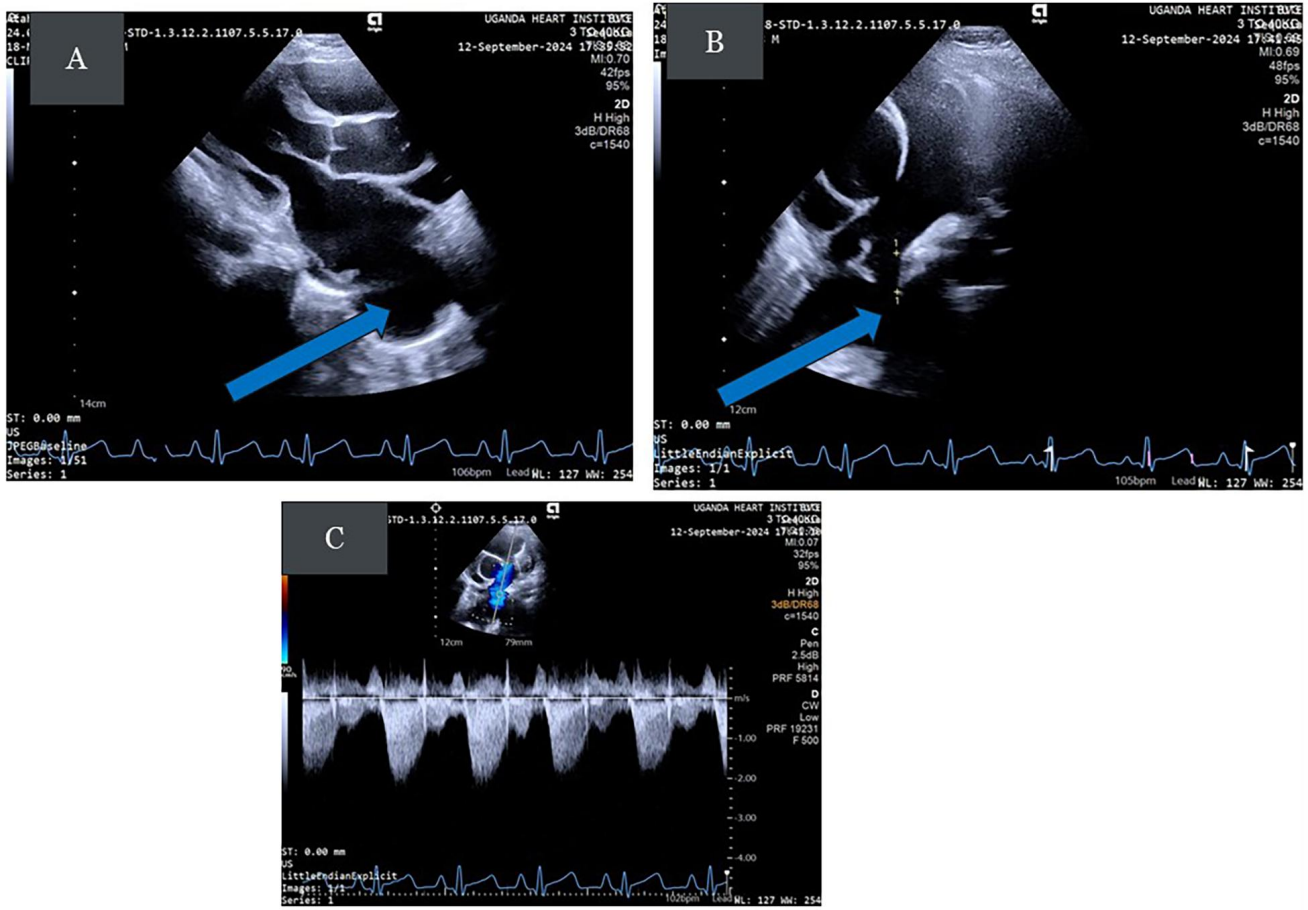
**(A)** Parasternal long-axis (PLAX) view demonstrating the dilated LA and a large aneurysm posterior to the left atrium (bold arrow). **(B)** Modified parasternal short-axis view showing a dilated RPA and a fistula connecting the RPA (small arrows) to the aneurysm (bold arrow). The pulmonary artery may dilate due to either increased flow or increased pulmonary artery pressure. **(C)** Doppler signal tracing showing the typical systolic, diastolic wave forms with a wave reversal.

The chest X-ray demonstrated a dilated left ventricle (LV) with a double density within the cardiac silhouette on the lower right half, merging superiorly with the right pulmonary artery shadow, suggesting an aneurysmal entry zone of the RPA to LA (see Figure 1A). The electrocardiogram (ECG) revealed sinus rhythm with a rightward QRS axis and biventricular hypertrophy (see Figure 1B). Transthoracic echocardiogram (TTE) revealed dilated LA and LV, a small patent foramen ovale (PFO), a large aneurysm connected to the roof of the LA, and a fistula connecting a dilated RPA to the aneurysm with a typical Doppler signal (see Figures 2A–C) (Supplementary Videos 1a–d). An agitated saline bubble contrast echocardiogram demonstrated complete rapid filling of the left heart chambers within four heart beats. A contrast-enhanced chest computed tomography (CT) angiogram confirmed the RPA to LA fistula. The patient was scheduled for elective transcatheter occlusion.

**Figure 3:**
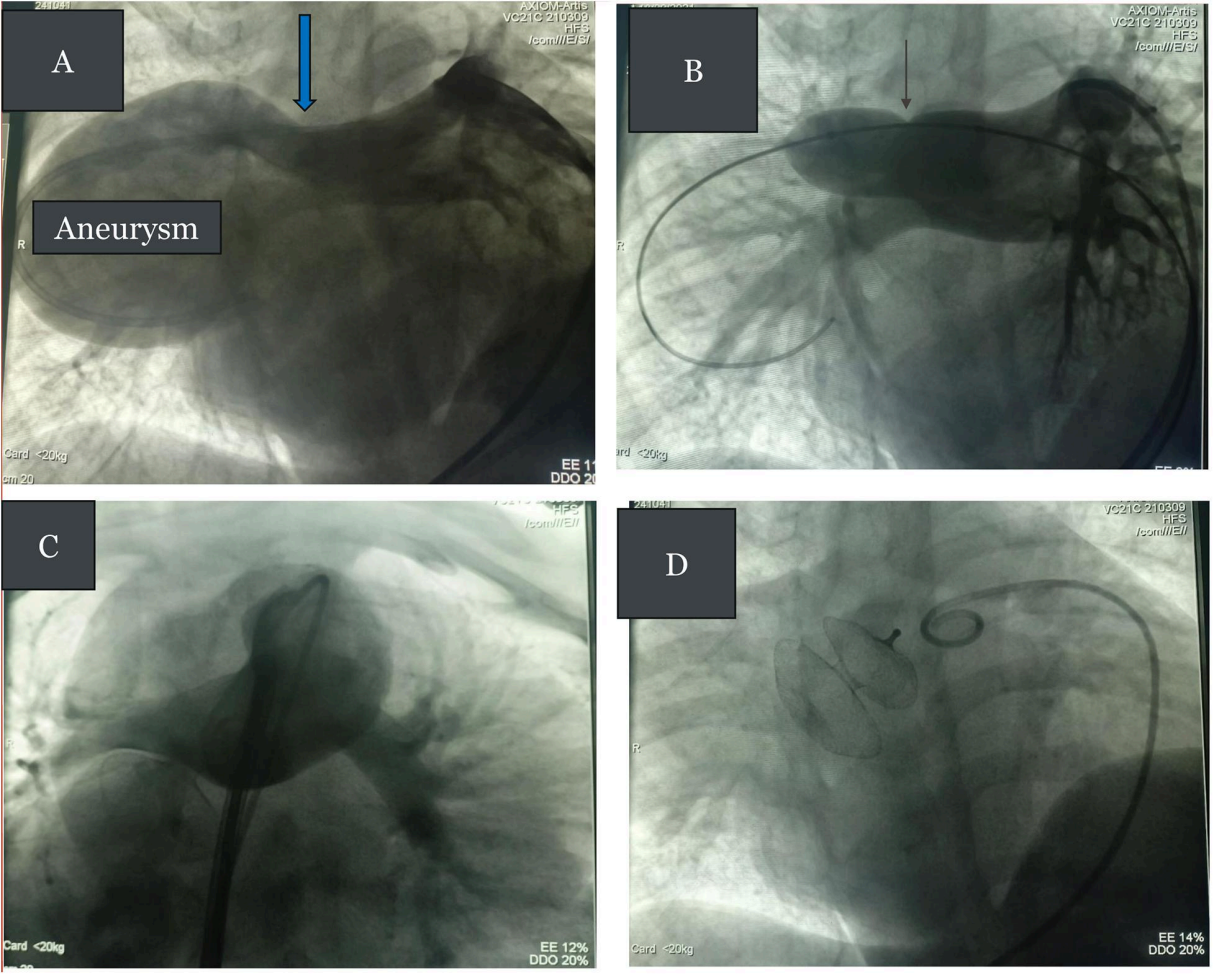
**(A)** RPA angiogram in the right anterior oblique (RAO) projection showing a fistula connecting the RPA to the LA (blue arrow) with an intervening aneurysm. One catheter tip is in the proximal RPA and one the LA. **(B)** Balloon test occlusion of the RPA to LA fistula with waist formation (black arrow). Note normal flow to the RPA branches. **(C)** Check RPA angiogram before release of occluder device showing normal flow to the RPA branches. **(D)** Final appearance of the device after its release in the fistula.

Cardiac catheterization was performed under general anesthesia with transesophageal echo (TEE) guidance. Bilateral femoral venous access and right femoral arterial access were obtained. The following preliminary pressure data (mm Hg, recorded as systolic/diastolic/mean) were obtained: right atrium (RA) = 10/4/6; left atrium (LA) = 15/7/10; femoral artery: 87/55/66; right ventricle (RV): 22/4; and right pulmonary artery (RPA): 21/11/14. Systemic oxygen saturation was 72%. An RPA angiogram using a 5 Fr pigtail catheter confirmed a fistulous connection between the superior aspect of the RPA and a large aneurysm that connected directly to the LA roof (see Figure 3A, Supplementary Videos 2a–d). Balloon test occlusion of the fistula with a Tyshak II 18 mm × 30 mm balloon over a 0.035" Amplatzer extra-stiff wire, placed and looped in the LA, showed a narrowest diameter of 12 mm with normalization of oxygen saturations (see Figure 3B). Blood flow to all RPA branches was preserved. The fistula was closed by placing a 9 F delivery sheath over the wire to the LA and using a 15 mm Occlutech Figulla Flex II occluder, with the LA disc in the LA aspect of the aneurysm and the RA disc protruding into the native RPA but not obstructing it (see Figures 3C,D). Post-procedure pressure measurements were as follows (mmHg, systolic/diastolic/mean): femoral artery 88/55/66; pulmonary artery (PA): 37/15/22; oxygen saturations = 100%. Intraprocedural TEE showed good device position with minimal residual flow through the device, and the device was released. Hemostasis was obtained using manual compression.

**Figure 4:**
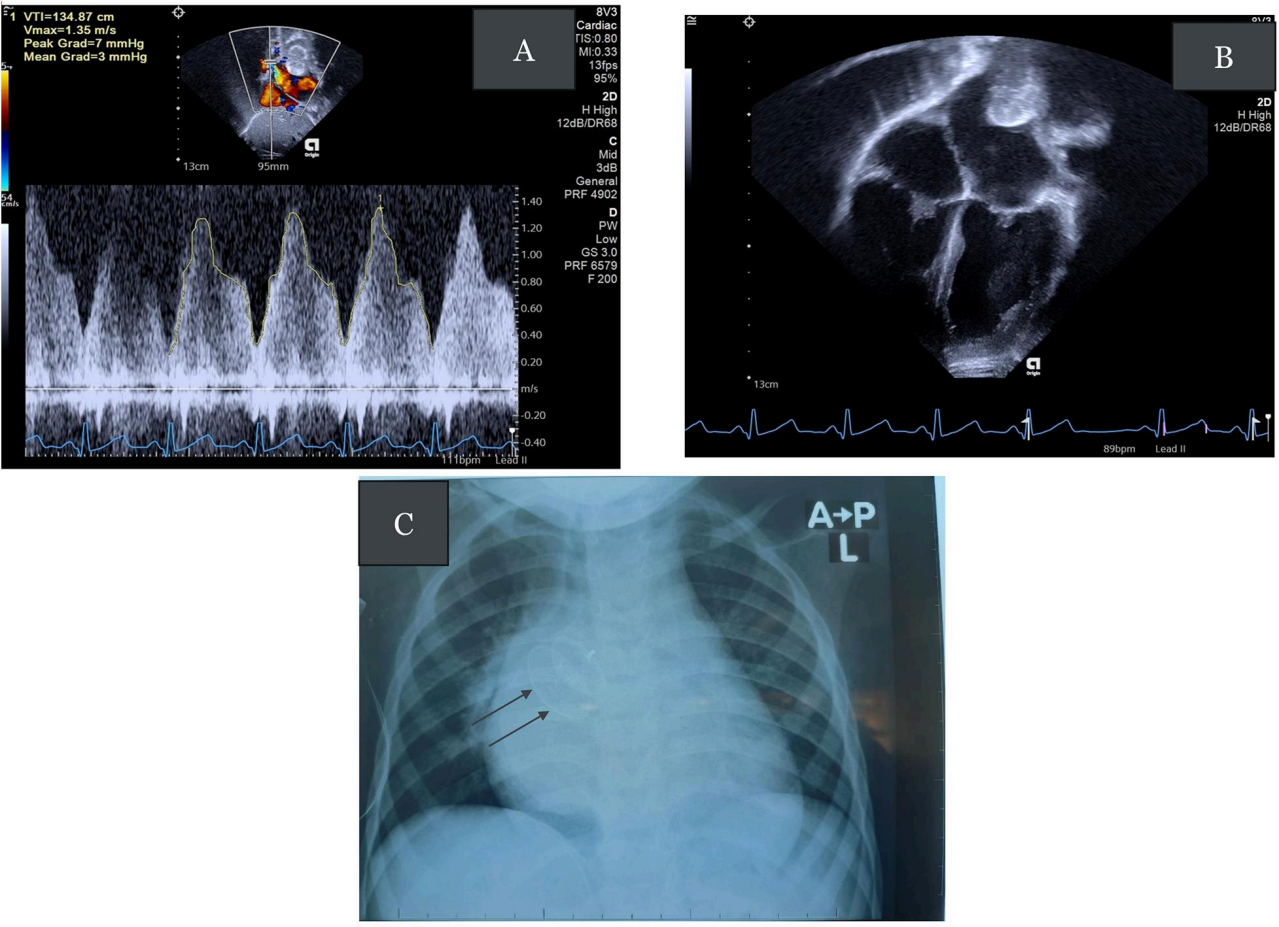
**(A)** Continuous wave Doppler tracing showing mild flow acceleration in the RUPV with a mean pressure gradient (PG) of 3 mmHg. **(B)** Apical four-chamber view showing the device posterosuperior to the LA. **(C)** Post-procedure chest radiograph showing satisfactory device position (black arrows).

The patient was started on low-molecular-weight heparin for 5 days and warfarin for 6 months. A repeat transthoracic echo the following day revealed a good device position without a residual shunt. There was flow acceleration in the right upper pulmonary vein (RUPV) with a mean gradient of 3 mmHg (see Figures 4A, B, and Supplementary Videos 3a–c). A repeat chest radiograph revealed good device position (see Figure 4C). The patient developed fever in the post-procedure period that resolved within 2 days. The patient remained hospitalized for 14 days to optimize the international normalized ratio (INR) and was then discharged. Follow-up evaluations were unremarkable, with the latest review9 months following the procedure showing no gradient in the RUPV (see Supplementary Figure 1 for the care timeline).

## Discussion

A pulmonary artery to left atrial fistula is a rare cyanotic CHD. The embryologic basis of this anomaly is thought to be incomplete degeneration of the septum between the arterial and venous plexus of the pulmonary vascular bed. It may also result from the formation of thin-walled sacs because of a defect in the capillary loops. A pulmonary vein connected to such a fistula is then resorbed into the LA during development, forming a PA–LA fistula (4). Agenesis of a lung lobe with the subsequent absence of its pulmonary capillary bed is thought to be another possible explanation (4).

Four anatomic types (I–IV) of PA–LA fistulas have been described based on the involvement of the pulmonary veins and the branching pattern of the right pulmonary artery (2, 3). In Type I, the commonest form (accounting for about one-third of all cases) (5), the pulmonary venous return and the RPA branching pattern are normal, except for a fistulous channel connecting the RPA to the left atrium forming an aneurysmal sac. This type corresponds to our case. In Type II, the right lower branch of the RPA connects directly to the LA, forming an aneurysmal sac in the place of an absent right lower pulmonary vein. This type is typically associated with right lung abnormalities such as the absence of one or more lobes (6). In Type III, all the right- and left-sided pulmonary veins drain into the aneurysmal sac that connects the RPA to the LA, whereas in Type IV, it is only the right-sided pulmonary veins that drain into this aneurysmal sac with the left-sided pulmonary veins connecting normally to the LA. Common associated findings include an interatrial communication (ASD or PFO). Anomalies of the right lung, such as the absence of a lower or middle lobe, sequestration, and diverticulum of the right main bronchus, have also been described (7, 8).

This defect affects more boys than girls (with a male to female ratio of 3:1) (5), and right-sided fistulas are much more common than left-sided ones. Clinical features are dependent on the size of the fistula, the magnitude of right-to-left shunt, and the age at presentation. In infancy, cyanosis and heart failure are common symptoms, whereas older children and adults may present with dyspnea, cyanosis, and neurologic symptoms. Precordial examination is often unremarkable, and an audible murmur may be heard in some cases. The chest radiograph may reveal an abnormal cardiac shadow. A high index of suspicion is required for accurate diagnosis. Transthoracic echocardiography may reveal an enlarged LA and the fistulous connection. In the absence of an associated intracardiac shunt, agitated saline contrast echocardiogram is often diagnostic, showing contrast in left-sided cardiac chambers after three beats (9, 10). CT typically delineates the anomalous connection. In this case, because the findings on transthoracic echocardiography were so typical, additional imaging with contrast echocardiography and computed tomography could have been omitted due to their associated risks (11, 12), leaving them for cases of uncertainty or pre-procedural planning. Magnetic resonance imaging (MRI) is also an ideal alternative when feasible.

Therapeutic options include surgical ligation or transcatheter closure of the fistulous tract using coils or various occluder devices at the narrowest point without causing obstruction to pulmonary venous flow. Depending on the available occluder devices (patent ductus arteriosus (PDA), muscular ventricular septal defect (VSD), and ASD occluders have been used) (13), either a transseptal or transpulmonary approach could be used. The goal is to always have the larger disc of the device on the pulmonary arterial side of the fistula. During the procedure, we ensured a large proximal diameter of the device implanted by oversizing the device used to prevent any dislodgment. The transseptal approach is relatively more straightforward, avoids multiple turns, and offers better wire support, whereas the transpulmonary approach eliminates the need for transseptal puncture in patients without an interatrial communication (14). With the transseptal approach, a wire loop can be created and a duct occluder device can be deployed from the PA side, which is especially useful when the available long delivery sheaths are relatively stiff. We elected to use the transpulmonary approach. The only available device was an Occlutech ASD occluder, and the Occlutech ASD delivery sheath was relatively soft, easily making the curves to the RPA without kinking. Although the ASD device is relatively bulky and can potentially cause obstruction in small children, we did not observe any in the early follow-up period as the gradient in the RUPV had resolved at the last clinic visit. The Occlutech Figulla Flex II occluder 15 mm device used has a distal disc (LA) size of 30 mm and proximal disc size of 26 mm. This oversizing essentially ensures more than double the narrowest size measured (12 mm) by balloon sizing on the pulmonary artery side, to prevent any dislodgment or embolization.

Standard therapy for atrial septal device implantation involves full heparinization during the procedure, followed by antiplatelet drugs for 6 months. However, we chose to use anticoagulation with warfarin, recognizing the thrombosis risk posed by the aneurysmal dilatation at the entry point of the fistula into left atrium. Typically, a TEE or repeat CT is needed to confirm aneurysm resolution without thrombus formation. This was not performed in this case because of a breakdown in the CT service in the immediate follow-up period.

## Conclusion

Pulmonary artery to LA fistula is a rare cause of cyanosis. A high index of suspicion is necessary, with accurate diagnosis requiring a combination of contrast echocardiography and CT imaging. Transcatheter occlusion of the fistula, using various types of available devices, is now the standard of care, offering excellent results.

## Data Availability

The original contributions presented in the study are included in the article/Supplementary Material; further inquiries can be directed to the corresponding author.
