# Supplementary material for: Case Report: Transcatheter occlusion of a rare pulmonary artery to left atrium fistula using an atrial septal defect occluder device
Source: Front Cardiovasc Med. 2026 Mar 2;12:1698642. doi: 10.3389/fcvm.2025.1698642 (PMC12989974; doi:10.3389/fcvm.2025.1698642)
Supplement: Supplementary file 1 [file Presentation1.pptx]

## Slide 1
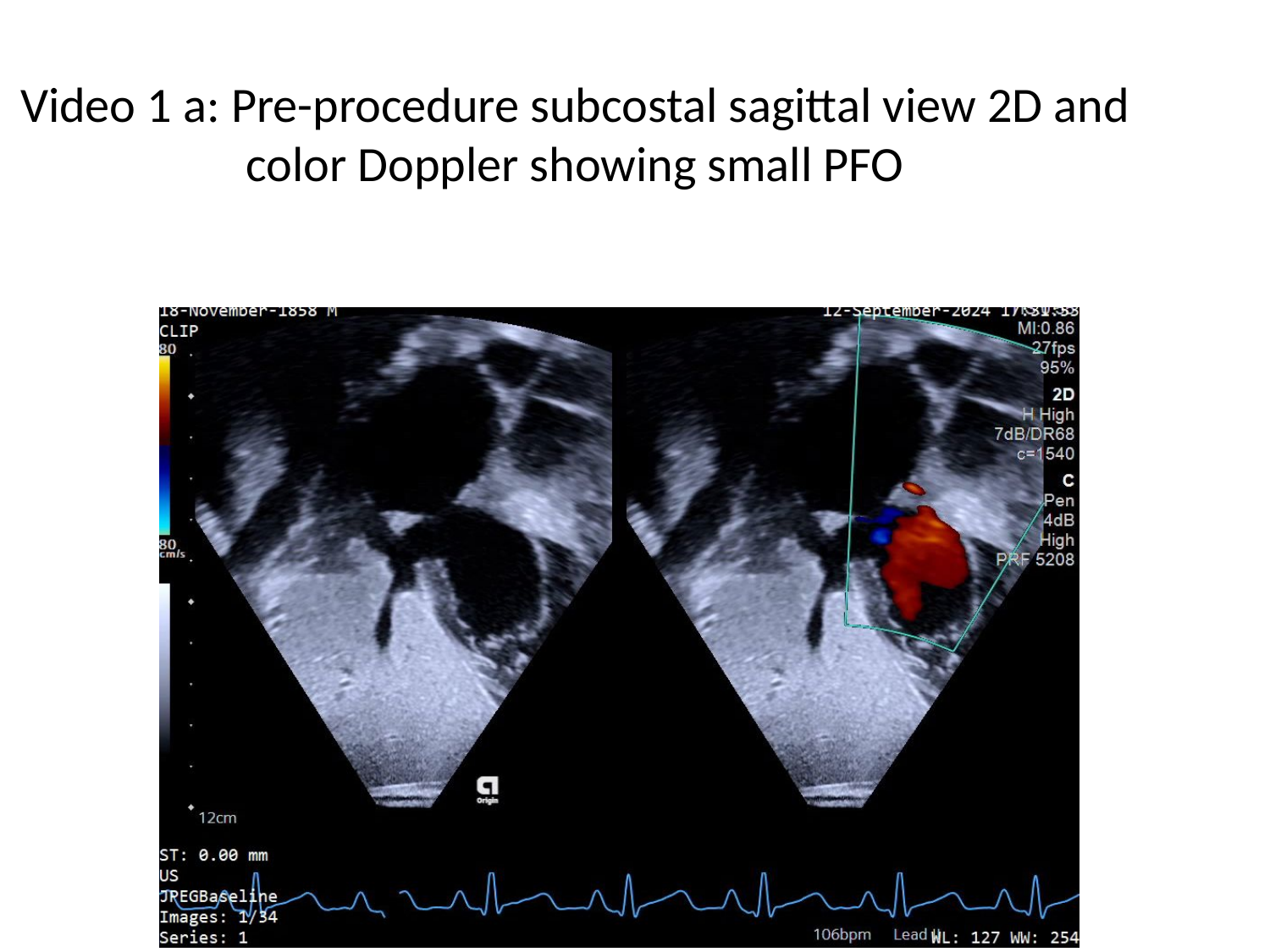

# Video 1 a: Pre-procedure subcostal sagittal view 2D and color Doppler showing small PFO

## Slide 2
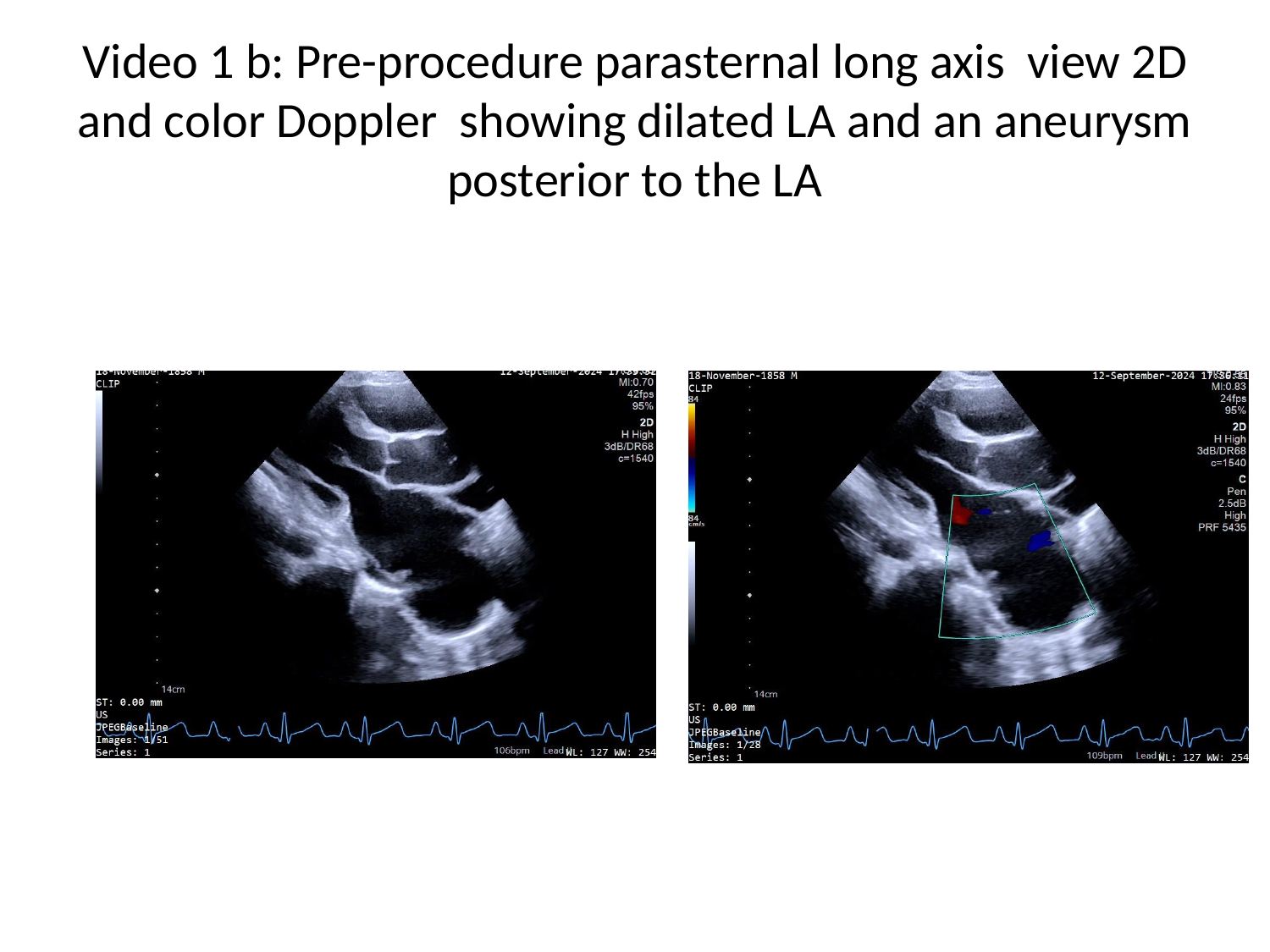

# Video 1 b: Pre-procedure parasternal long axis view 2D and color Doppler showing dilated LA and an aneurysm posterior to the LA

## Slide 3
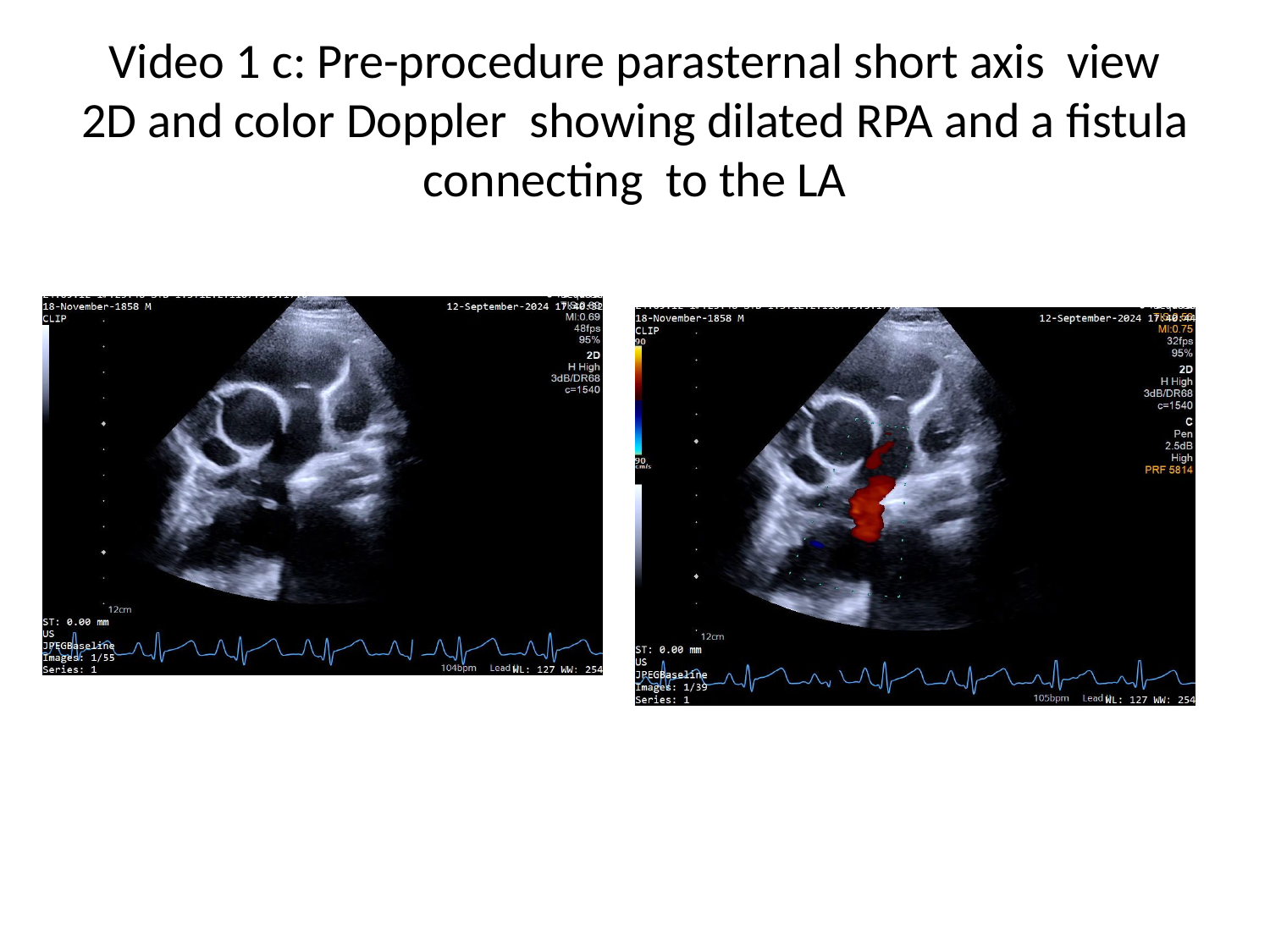

# Video 1 c: Pre-procedure parasternal short axis view 2D and color Doppler showing dilated RPA and a fistula connecting to the LA

## Slide 4
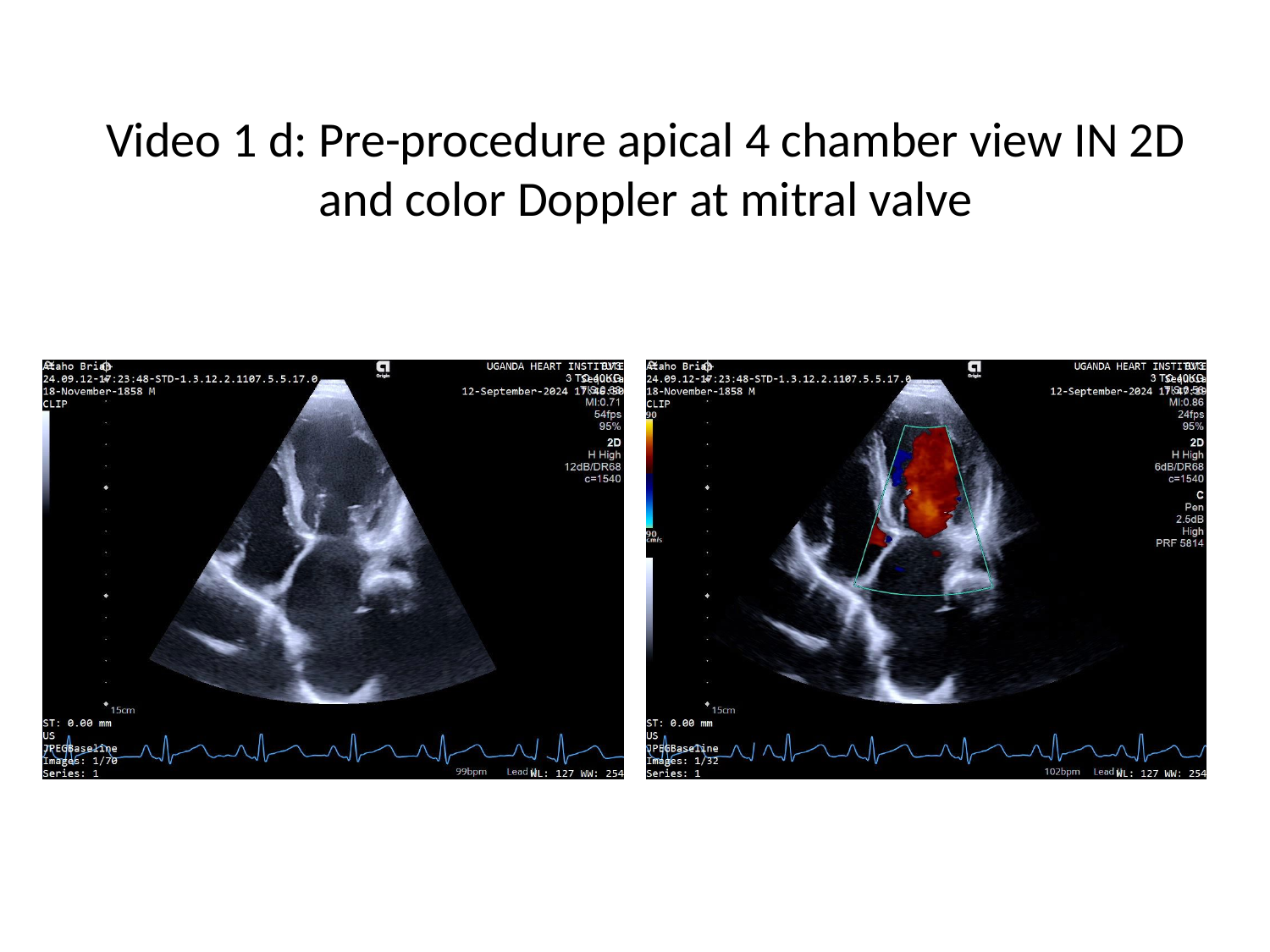

# Video 1 d: Pre-procedure apical 4 chamber view IN 2D and color Doppler at mitral valve
