# Supplementary material for: Case Report: Transcatheter occlusion of a rare pulmonary artery to left atrium fistula using an atrial septal defect occluder device
Source: Front Cardiovasc Med. 2026 Mar 2;12:1698642. doi: 10.3389/fcvm.2025.1698642 (PMC12989974; doi:10.3389/fcvm.2025.1698642)
Supplement: Supplementary file 2 [file Presentation2.pptx]

## Slide 1
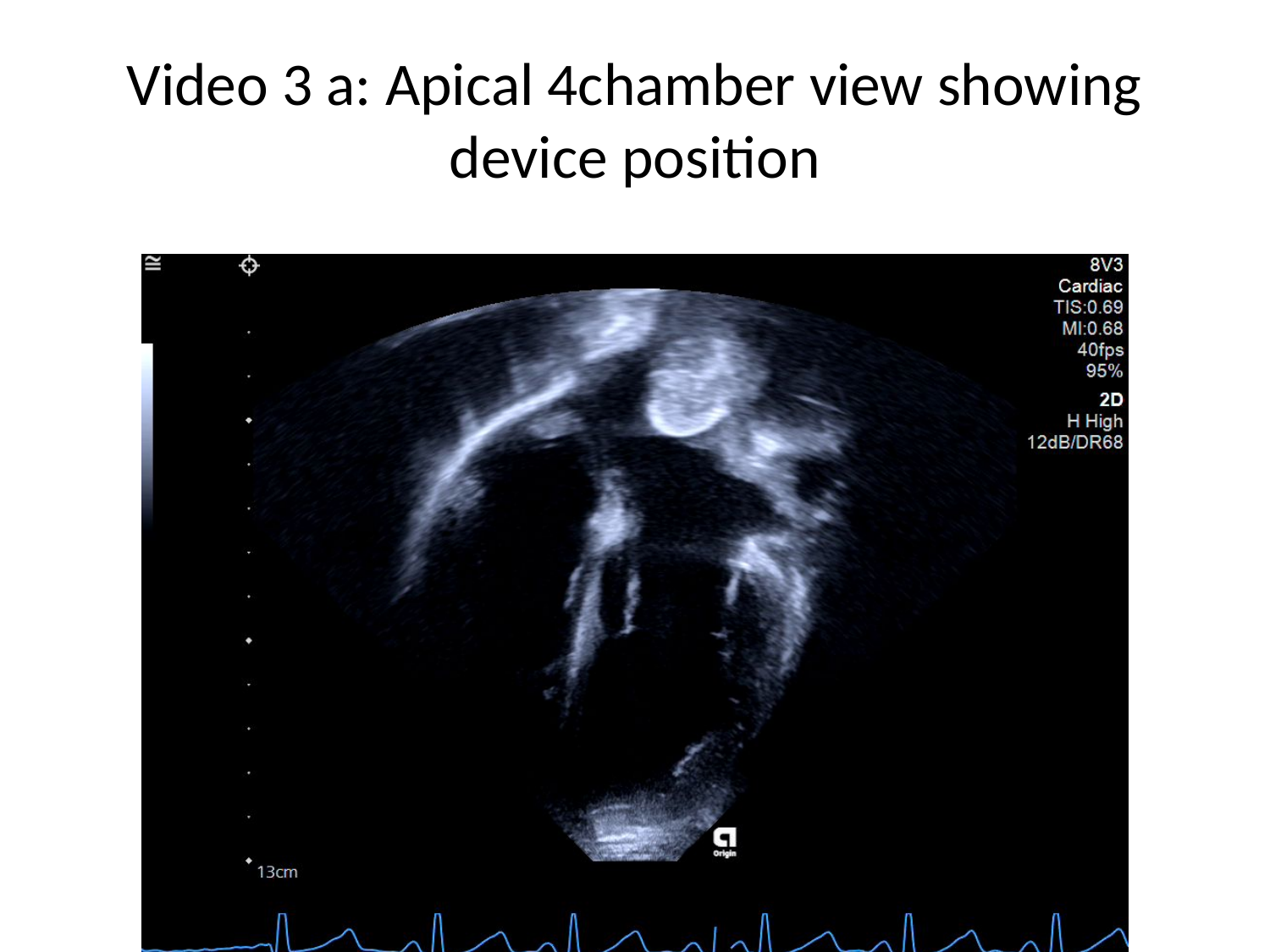

# Video 3 a: Apical 4chamber view showing device position

## Slide 2
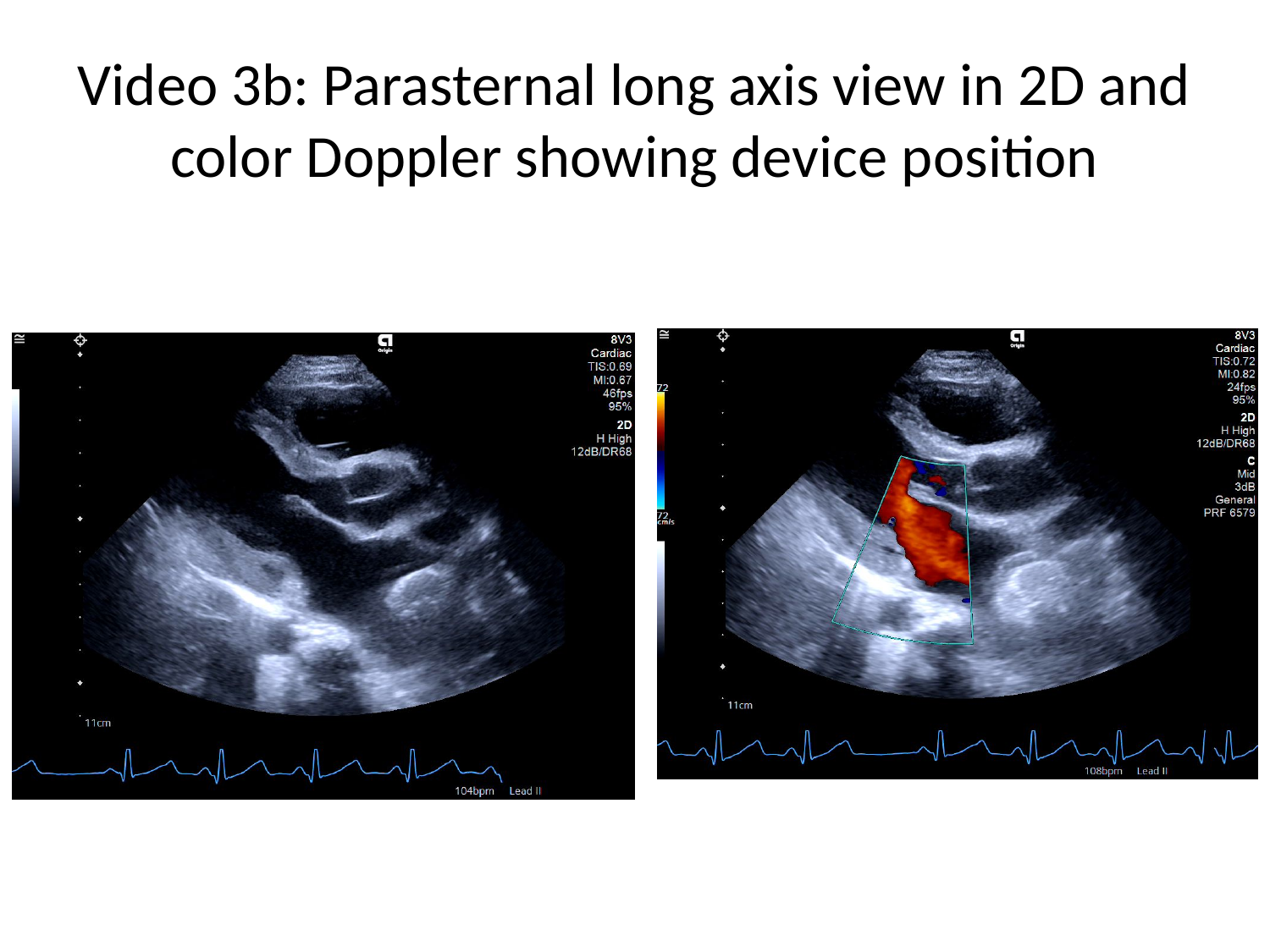

# Video 3b: Parasternal long axis view in 2D and color Doppler showing device position

## Slide 3
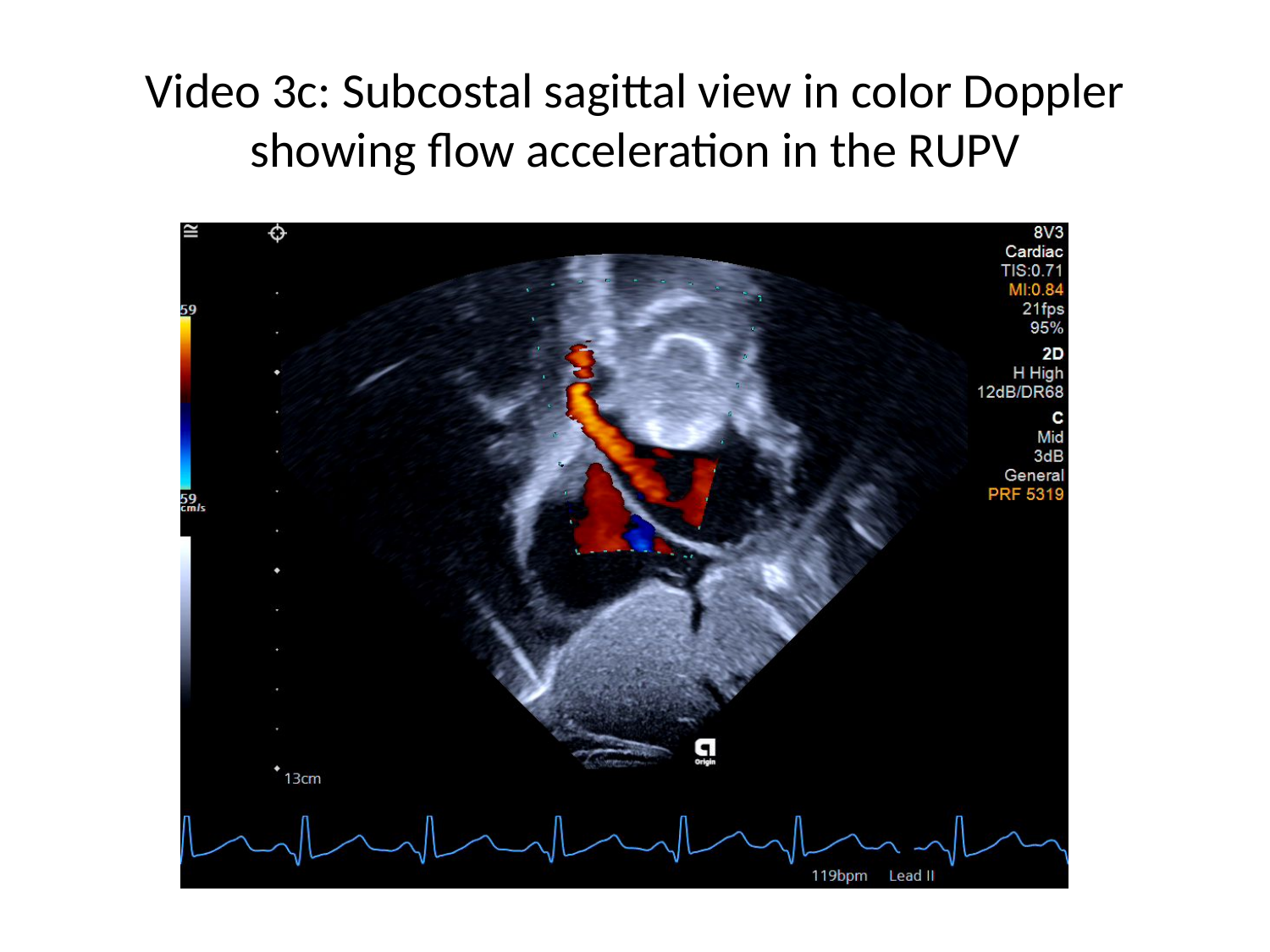

# Video 3c: Subcostal sagittal view in color Doppler showing flow acceleration in the RUPV
