# Supplementary figures and images for: Case Report: Transcatheter occlusion of a rare pulmonary artery to left atrium fistula using an atrial septal defect occluder device
Source: Front Cardiovasc Med. 2026 Mar 2;12:1698642. doi: 10.3389/fcvm.2025.1698642 (PMC12989974; doi:10.3389/fcvm.2025.1698642)

## Slide 1
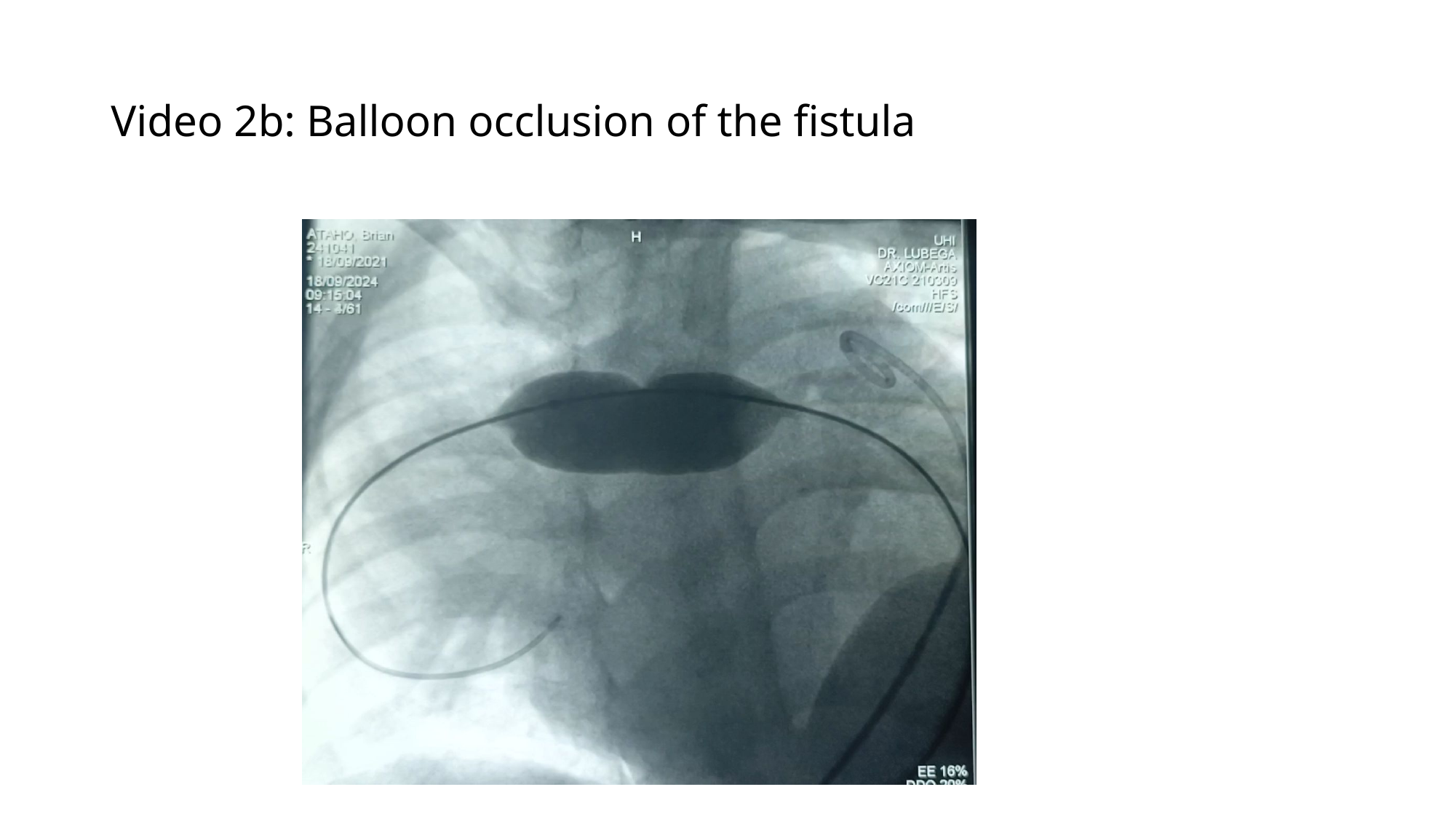

# Video 2b: Balloon occlusion of the fistula

Supplement: Supplementary file 3 [file Presentation3.pptx]

## Slide 1
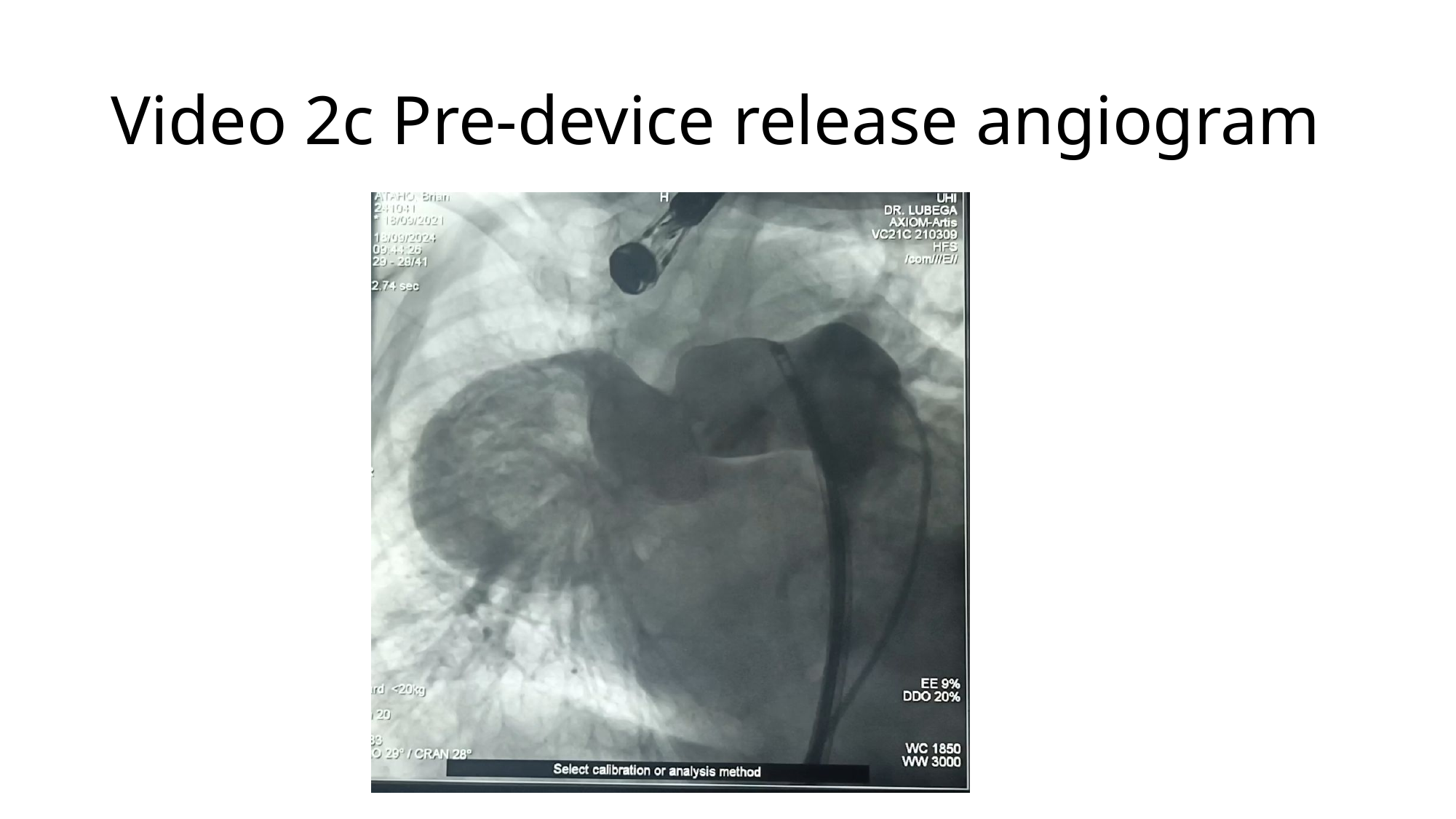

# Video 2c Pre-device release angiogram

Supplement: Supplementary file 4 [file Presentation4.pptx]

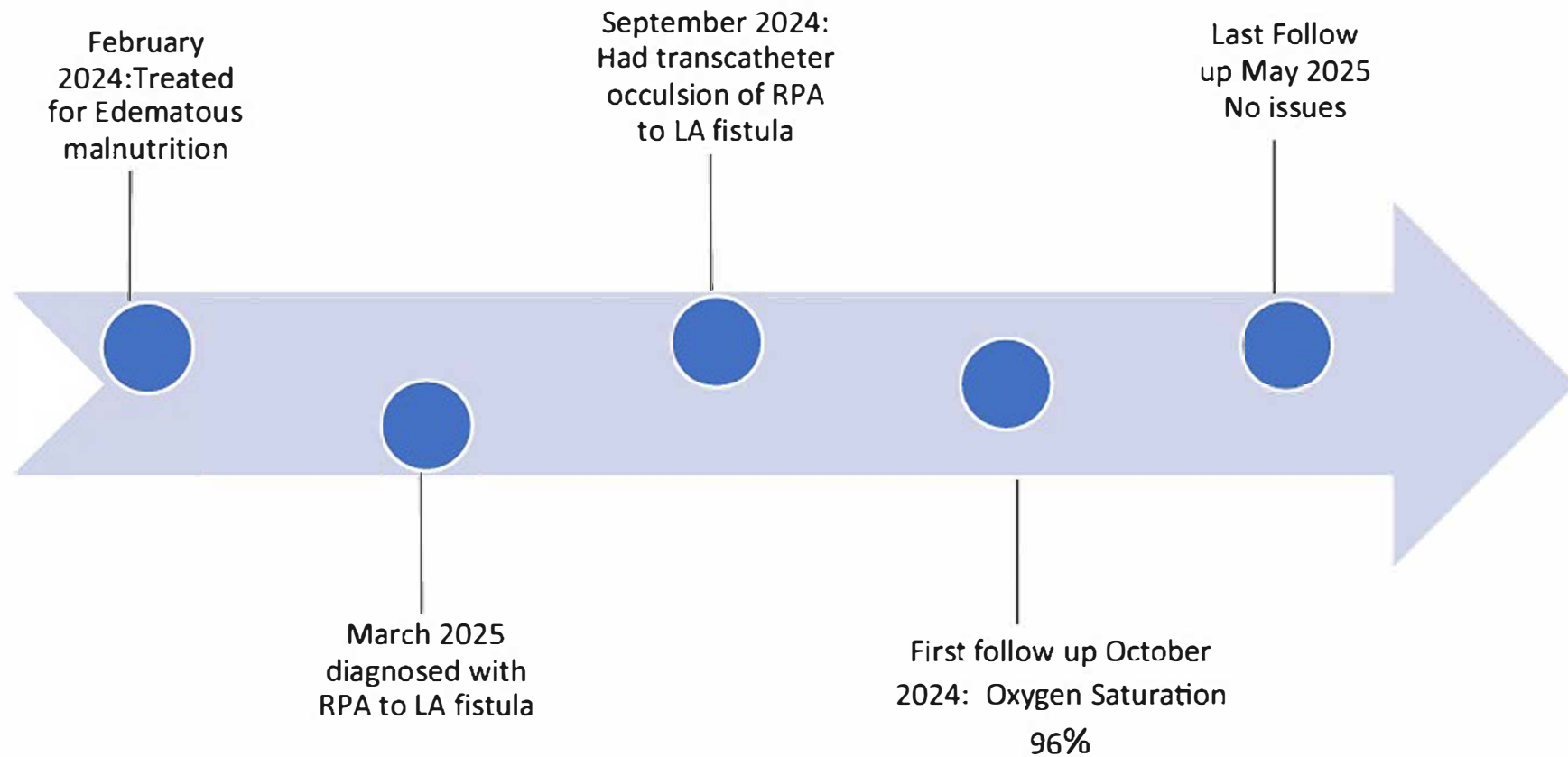

Supplementary Figure 1: Care timeline

Supplement: Supplementary file 5 [file Image1.pdf]
